# Pregnancy-Associated Plasma Protein A Induces Inflammatory Cytokine Expression by Activating IGF-I/PI3K/Akt Pathways

**DOI:** 10.1155/2019/8436985

**Published:** 2019-09-10

**Authors:** Weiping Li, Hongwei Li, Li Zhou, Zijian Wang, Bing Hua

**Affiliations:** ^1^Department of Cardiology, Cardiovascular Center, Beijing Friendship Hospital, Capital Medical University, Beijing 100050, China; ^2^Beijing Key Laboratory of Metabolic Disorder Related Cardiovascular Disease, Beijing 100069, China; ^3^Department of Internal Medicine, Medical Health Center, Beijing Friendship Hospital, Capital Medical University, Beijing 100050, China

## Abstract

Pregnancy-associated plasma protein A (PAPP-A) was previously reported to be an inflammatory biomarker and a prognostic marker of acute coronary syndrome (ACS) and involved in the process of atherosclerosis and plaque rupture. However, the role of PAPP-A in inflammation is poorly understood. In this study, we aimed to investigate the role of PAPP-A in macrophage activation and inflammatory cytokine production. RAW264.7 macrophages were treated with or without PAPP-A. Reverse-transcriptase quantitative real-time PCR (RT-qPCR) and Western blot were performed to detect gene and protein expressions. The concentration of monocyte chemoattractant protein-1 (MCP-1), tumor necrosis factor-*α* (TNF-*α*), and interleukin-6 (IL-6) in culture supernatants was determined by ELISA. Results showed that PAPP-A significantly stimulated the expression of MCP-1, TNF-*α*, and IL-6 at both transcriptional and translational levels in a dose-dependent and time-dependent manner. The secretion of these inflammatory cytokines by macrophages was also increased after PAPP-A treatment. Moreover, PAPP-A activated the IGF-I/PI3K/Akt signaling pathway in macrophages. The PAPP-A-mediated upregulation of MCP-1, TNF-*α*, and IL-6 mRNA and protein levels were strongly inhibited by PI3K inhibitors or IGF-IR siRNA, indicating that the upregulation of MCP-1, TNF-*α*, and IL-6 could involve the IGF-I/PI3K/Akt pathway. Together, this study demonstrates that PAPP-A activates the macrophage signaling pathway (IGF-I/PI3K/Akt), which drives the expression and production of inflammatory cytokines known to contribute to the initiation and progression of ACS. These findings indicate that PAPP-A may play a proinflammatory role in the pathophysiology of ACS and serve as a potential therapeutic target.

## 1. Introduction

Atherosclerosis, a complex inflammatory response to chronic endothelial injury, involves several cell types and multiple cytokines, growth factors, and enzymes [1]. Increasing evidences support a central role of inflammation in atherosclerosis progression and complication and acute coronary syndrome (ACS) development. The characteristic components of atherosclerotic plaque are macrophages, which can produce various chemokines, proinflammatory cytokines, and inflammatory mediators, such as monocyte chemoattractant protein-1 (MCP-1), interleukin-6 (IL-6), interleukin-1*β* (IL-1*β*), and tumor necrosis factor-*α* (TNF-*α*). Thus, macrophages may contribute to atherosclerosis development and ACS pathogenesis.

Pregnancy-associated plasma protein A (PAPP-A) is a zinc-binding metalloproteinase protein and involved in the insulin-like growth factor (IGF) system [2]. In 2001, Conover et al. first reported that PAPP-A was abundantly expressed in coronary plaque cells and the extracellular matrix of ruptured and eroded unstable plaques from patients who died from sudden cardiac causes. In plaques with large lipid cores and cap rupture, positive PAPP-A staining appeared mostly in the inflammatory shoulder region and in the areas surrounding the lipid core and was located with CD68-positive cells [3]. PAPP-A has also been detected in ruptured and eroded plaques from human arterial specimens with the strongest staining observed in the inflammatory areas containing activated macrophages and smooth muscle cells, which indicated PAPP-A might be associated with plaque instability and rupture [4, 5]. Recent studies found that the elevated PAPP-A levels in the supernatant of cultured HUVECs were associated with a reduction in nitric oxide (NO), which is important for maintaining normal endothelial function, thus amplifying atherosclerotic disease progression [6]. It has been demonstrated that PAPP-A induced a prothrombotic phenotype in endothelial cells since it promoted the expression of functionally active tissue factor (TF) in these cells [7]. Furthermore, previous studies from our group and other investigators have found that an increase in PAPP-A plasma levels in patients with stable coronary disease or ACS was associated with a higher risk of developing future acute cardiovascular events [8–11]. Notably, several animal model studies have shown the involvement of PAPP-A in atherosclerotic lesion development [12–15]. Compared with ApoE knockout (KO) mice, the ApoE/PAPP-A double KO mice had significantly reduced aortic plaque burden. Targeted inhibition of PAPP-A activity reduced aortic plaque burden and plaque complexity in ApoE KO mice on a high-fat diet. These results suggest that PAPP-A may contribute to plaque instability and rupture.

Despite these findings, the mechanism underlying PAPP-A-mediated plaque rupture and major cardiovascular events remains unclear. Our current study is aimed at filling this knowledge gap. In vitro, PAPP-A has been shown to cleave inhibitory IGF binding protein-4 (IGFBP-4) thereby increasing local IGF bioavailability and receptor activation [16, 17]. Free IGF-I can thus interact with its membrane receptor (IGF-IR) and activate the phosphatidylinositol-3 (PI3K) and Akt kinase (Akt) signaling pathways, which can stimulate the expression of inflammatory mediators MCP-1, TNF-*α*, and IL-6 in activated macrophages [18].

Based on these studies, we hypothesized that PAPP-A might increase the expression of MCP-1, TNF-*α*, and IL-6 in RAW264.7 macrophages through the IGF-I-mediated signaling pathway (IGF-I/PI3K/Akt), thereby promoting inflammation and participating in the process of plaque disruption and ACS.

## 2. Materials and Methods

### 2.1. Reagents

PAPP-A was purchased from R&D Systems Inc. (Minneapolis, MN, USA, Catalog Number 2487-ZNF). LY294002 was purchased from Calbiochem (San Diego, CA, USA). The Dulbecco's modified Eagle medium (DMEM), fetal bovine serum (FBS), and TRIzol reagent were purchased from Invitrogen (Carlsbad, CA, USA). BCA protein assay kit was obtained from Pierce Chemical (Rockford, IL, USA). Primary antibodies against MCP-1 (ab25124), TNF-*α* (ab9739), IL-6 (ab208113), IGF-I (ab9572), IGF-IR (ab131476), IGFBP-5 (ab125696), and *β*-actin (ab8227) were obtained from Abcam (Cambridge, UK). Primary antibodies against p85 (#4292S), phospho-p85 (#4228S), Akt (#9272S), and phospho-Akt (#9271S) were purchased from Cell Signaling Technology (Danvers, MA, USA). Unless otherwise noted, all other chemicals were obtained from Sigma-Aldrich (St. Louis, MO, USA). ELISA kits for MCP-1, TNF-*α*, and IL-6 in cell culture supernatants were purchased from R&D Systems Inc. (Minneapolis, MN, USA). ELISA kit for free IGF-I was purchased from BlueGene Biotech (Shanghai, China).

### 2.2. Cell Lines

RAW264.7 macrophages (mouse origin) were purchased from the American Type Culture Collection (ATCC, Rockville, MD, USA). Macrophages were grown and cultured in DMEM supplemented with 10% fetal bovine serum, penicillin (100 U/mL), and streptomycin (100 mg/mL) at 37°C under a humidified atmosphere containing 95% air and 5% CO_2_. To perform gene expression, protein expression, and ELISA analysis, macrophages were seeded in 48-well plates (2.5 × 10^5^ cells/mL, 300 *μ*L per well) or in 6-well plates (2.5 × 10^5^ cells/mL, 2 mL per well). To examine whether PAPP-A upregulates the expression and production of MCP-1, TNF-*α*, and IL-6, the cells were incubated with various concentrations (50, 100, and 200 ng/mL) of PAPP-A for 24 h or with 200 ng/mL PAPP-A for 0 h, 6 h, 12 h, and 24 h, respectively. To investigate whether the PAPP-A-mediated upregulation of chemokine and proinflammatory cytokines could be associated with the activation of the PI3K/Akt signaling pathway, we pretreated the RAW264.7 macrophages with LY 294002 (10 *μ*M), an inhibitor of the PI3K/Akt pathway, for 60 min before stimulation with PAPP-A (200 ng/mL). After treatment with various concentrations of PAPP-A for 24 h or with 200 ng/mL PAPP-A for different times or with LY 294002 and PAPP-A, the cells were harvested for quantitative real-time PCR and Western blot analysis, and the supernatants were collected for cytokine analysis.

### 2.3. RNA Isolation and Reverse-Transcriptase Quantitative Real-Time PCR (RT-qPCR)

The total RNA from different treated cells was isolated by using a TRIzol reagent (Invitrogen, Carlsbad, USA), according to the manufacturer's instructions. Reverse transcription was performed using M-MLV (GIBCO, Carlsbad, USA), and 100 ng of the RNA samples was collected from each culture condition. The quantitative real-time PCR was performed in the ABI PRISM 5700 sequence detector system using the ABI SYBR Green I kit (Applied Bio systems, Foster City, USA) at the following conditions: 50°C for 2 min and 95°C for 10 min, followed by 40 cycles of 95°C for 20 s and 60°C for 30 s, followed by a melt curve stage of 95°C for 15 s, 60°C for 30 s, and 95°C for 15 s. The primers used in the study are listed in Table 1. Melt curve analyses of all real-time PCR products were performed and shown to produce a single DNA duplex. Relative quantitation values were calculated using the 2^-*ΔΔ*CT^ method. The amount of each target gene was subsequently divided by the amount of internal control gene *β*-actin to obtain a normalized value. Three different experiments were performed for each experimental condition.

### 2.4. Western Blot

The cells were lysed with extraction buffer (50 mmol/L Tris-HCl pH 7.5, 0.5% Triton X-100, 0.5% NP40, 150 mmol/L NaCl, 2 mmol/L EDTA, 1 mmol/L NaF, 1 mmol/L PMSF, 1 mmol/L Na3VO4) and protease inhibitors. Cell lysates were centrifuged at 12,000 g for 5 min at 4°C. The protein concentration in the supernatants was determined by the Bradford Protein Assay (Bio-Rad, Richmond, CA, USA). Protein samples (20–30 *μ*g) were separated on 10% SDS-PAGE gels and then transferred onto polyvinylidene fluoride (PVDF) membranes. The membranes were incubated with primary antibodies against MCP-1 (1 : 1000), TNF-*α* (1 : 1000), IL-6 (1 : 1000), IGF-I (1 : 1000), IGF-IR (1 : 1000), IGFBP5 (1 : 1000), p85 (1 : 2000), phospho-p85 (1 : 1000), Akt (1 : 2000), phospho-Akt (1 : 1000), and *β*-actin (1 : 2000) overnight at 4°C, then incubated with an HRP-conjugated secondary antibody for 1 h. The protein bands were detected with the Western Blotting Imaging System (Clinx Science Instruments Co., Shanghai, China). For the phospho-specific protein, we calculated the relative intensity for target proteins through dividing the absolute intensity of phosphorylated proteins by the absolute intensity of total target proteins or *β*-actin.

### 2.5. Enzyme-Linked Immunosorbent Assay (ELISA)

Cells were plated in six-well plates and treated as described above. Culture supernatants were collected and stored at −20°C until analysis. The concentrations of MCP-1, TNF-*α*, IL-6, and free IGF-I were determined by ELISA (R&D Systems, Minneapolis, MN, USA), following the manufacturer's procedures. Quantitative determinations in three different experiments were performed.

### 2.6. Transfection for IGF-IR Silencing

Small interfering RNA (siRNA) specific for mouse IGF-IR (catalog no. 4390771, Invitrogen) and nonsilencing as control siRNAs (catalog no. 4390843, Invitrogen) were ordered from Invitrogen. RAW264.7 macrophages (2 × 10^6^ cells/well) were transfected for 48 h using Lipofectamine 3000 (Invitrogen), according to the manufacturer's protocols.

### 2.7. Statistical Analysis

Statistical analysis was performed using SPSS 19.0 software. Data were expressed as the mean ± standard deviation (SD). Statistical differences among multiple groups were analyzed with a one-way analysis of variance (ANOVA) followed by Bonferroni's post hoc test. When two groups were compared, differences were analyzed using Student's *t*-test. A *P* value of <0.05 was considered statistically significant. All experiments were performed at least three times.

## 3. Results

### 3.1. PAPP-A Induced MCP-1, TNF-*α*, and IL-6 Expression in RAW264.7 Macrophages

**Figure 1:**
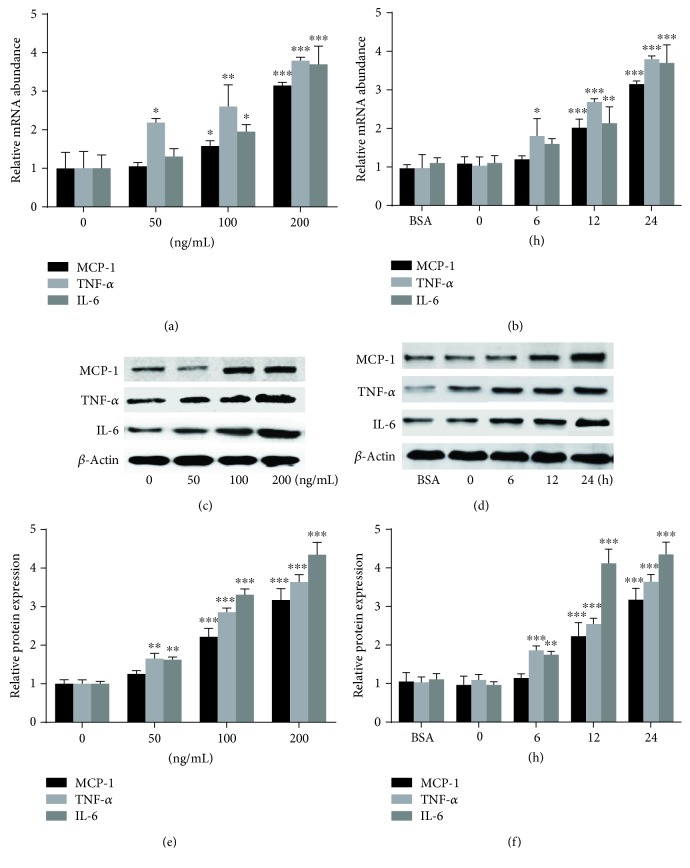
Dose-dependent and time-dependent effects of PAPP-A on MCP-1, TNF-*α*, and IL-6 expressions in RAW264.7 macrophages. (a, c, e) RAW264.7 macrophages were treated with various concentrations of PAPP-A for 24 h, respectively. (b, d, f) Cells were treated with 5 mg/mL BSA for 24 h or with 200 ng/mL PAPP-A for different durations, respectively. (a, b) MCP-1, TNF-*α*, and IL-6 mRNA expressions were measured by RT-qPCR. (c, d, e, f) MCP-1, TNF-*α*, and IL-6 protein expressions were measured by Western blot assays. All the results are expressed as the mean + SD from three independent experiments, each performed in triplicate. ^∗^*P* < 0.05 vs. baseline, ^∗∗^*P* < 0.01 vs. baseline, and ^∗∗∗^*P* < 0.001 vs. baseline.

In the present study, we first examined the effect of PAPP-A on the expression of MCP-1, TNF-*α*, and IL-6 in RAW264.7 macrophages by RT-qPCR and Western blot assays. RAW264.7 macrophages were incubated with various concentrations (50, 100, and 200 ng/mL) of PAPP-A for 24 h or 200 ng/mL PAPP-A for various times (6 h, 12 h, and 24 h). As shown in Figure 1, PAPP-A increased MCP-1, TNF-*α*, and IL-6 expressions at both transcriptional (Figures 1(a) and 1(b)) and translational (Figures 1(c)–1(f)) levels in a dose-dependent and time-dependent manner. The mRNA expression of TNF-*α* was increased by PAPP-A as early as 6 h, while MCP-1 and IL-6 were increased at 12 h. The protein expressions of TNF-*α* and IL-6 were significantly increased by PAPP-A at 6 h, while that of MCP-1 was increased at 12 h.

### 3.2. PAPP-A Induced MCP-1, TNF-*α*, and IL-6 Production in RAW264.7 Macrophages

**Figure 2:**
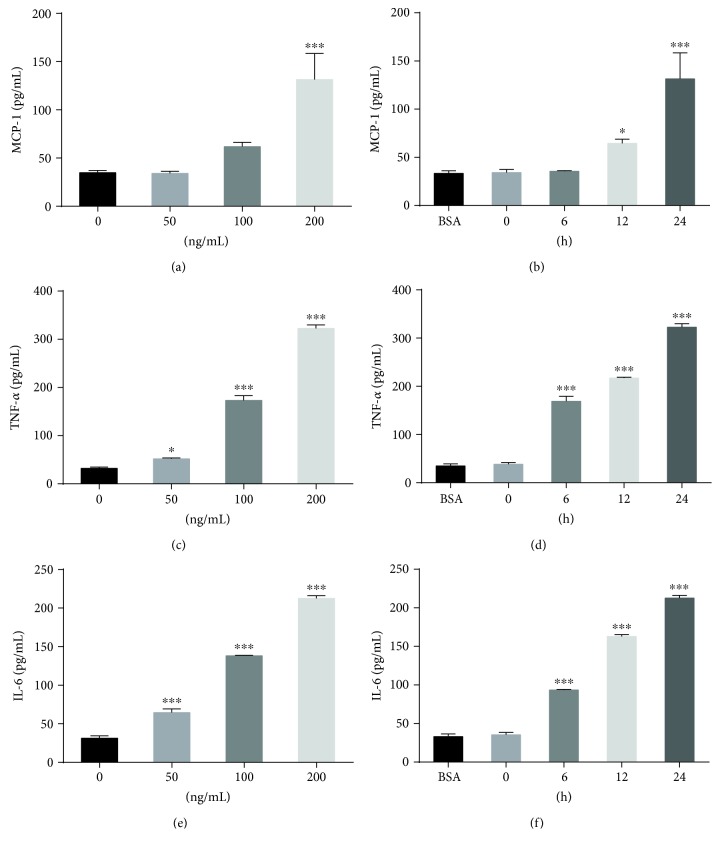
Dose-dependent and time-dependent effects of PAPP-A on MCP-1, TNF-*α*, and IL-6 productions in the supernatants of RAW264.7 macrophages. (a, c, e) RAW264.7 macrophages were treated with various concentrations of PAPP-A for 24 h, respectively. (b, d, f) Cells were treated with 5 mg/mL BSA for 24 h or with 200 ng/mL PAPP-A for different durations, respectively. MCP-1, TNF-*α*, and IL-6 concentrations were measured by ELISA assays. All the results are expressed as mean + SD from three independent experiments, each performed in triplicate. ^∗^*P* < 0.05 vs. baseline and ^∗∗∗^*P* < 0.001 vs. baseline.

The secretion of proinflammatory cytokines was tested by ELISA in the supernatants of RAW264.7 cells treated with PAPP-A. As shown in Figures 2(a), 2(c), and 2(e), the dose-response experiments confirmed that PAPP-A elicited a dose-dependent increase in the secretion of MCP-1, IL-6, and TNF-*α*. MCP-1, IL-6, and TNF-*α* levels in the supernatant increased by approximately 3-fold, 10-fold, and 7-fold, respectively, after PAPP-A (200 ng/mL) stimulation by 24 h. PAPP-A showed half maximal effectiveness on MCP-1 and TNF-*α* production at approximately 100 ng/mL, with maximal effects at approximately 200 ng/mL. The IL-6 level was increased significantly by PAPP-A at 50 ng/mL and then continued to elevate at a higher dose of PAPP-A. The time course of these proinflammatory cytokines in RAW264.7 cells under BSA, basal, and PAPP-A-stimulated conditions is presented in Figures 2(b), 2(d), and 2(f). Low proinflammatory cytokine levels were observed in RAW264.7 cultures under BSA or basal conditions after 24 hours. MCP-1 levels in culture supernatants increased at 12 h after stimulation with PAPP-A (200 ng/mL) and constantly increased by approximately 3-fold at 24 h. The levels of TNF-*α* and IL-6 increased at 6 h and peaked by approximately 10-fold and 7-fold at 24 h, respectively. These results suggest that PAPP-A may activate macrophages and promote inflammation.

### 3.3. PAPP-A Promoted the IGF-I/PI3-K/Akt Signaling Pathway in RAW264.7 Macrophages

PAPP-A specifically degrades insulin-like growth factor binding protein (IGFBP-) 4 and IGFBP-5, thereby releasing bioactive IGF-I to bind to cell surface IGF receptors [2, 17]. The binding of free IGF-I to its tyrosine kinase receptor (IGF-IR) leads to the activation of the PI3K/Akt signaling pathway [19, 20]. Because IGF-I has been found to stimulate IGFBP-5 mRNA expression in vitro as well as in vivo, an increased IGFBP-5 mRNA level can be used as an indicator of increased IGF signaling through the IGF-IR. An advantage for in vitro evaluation is that IGFBP-5 mRNA expression is upregulated for at least 24 h in response to IGF-I receptor activation in contrast with the transient changes in intracellular signaling intermediates [15].

**Figure 3:**
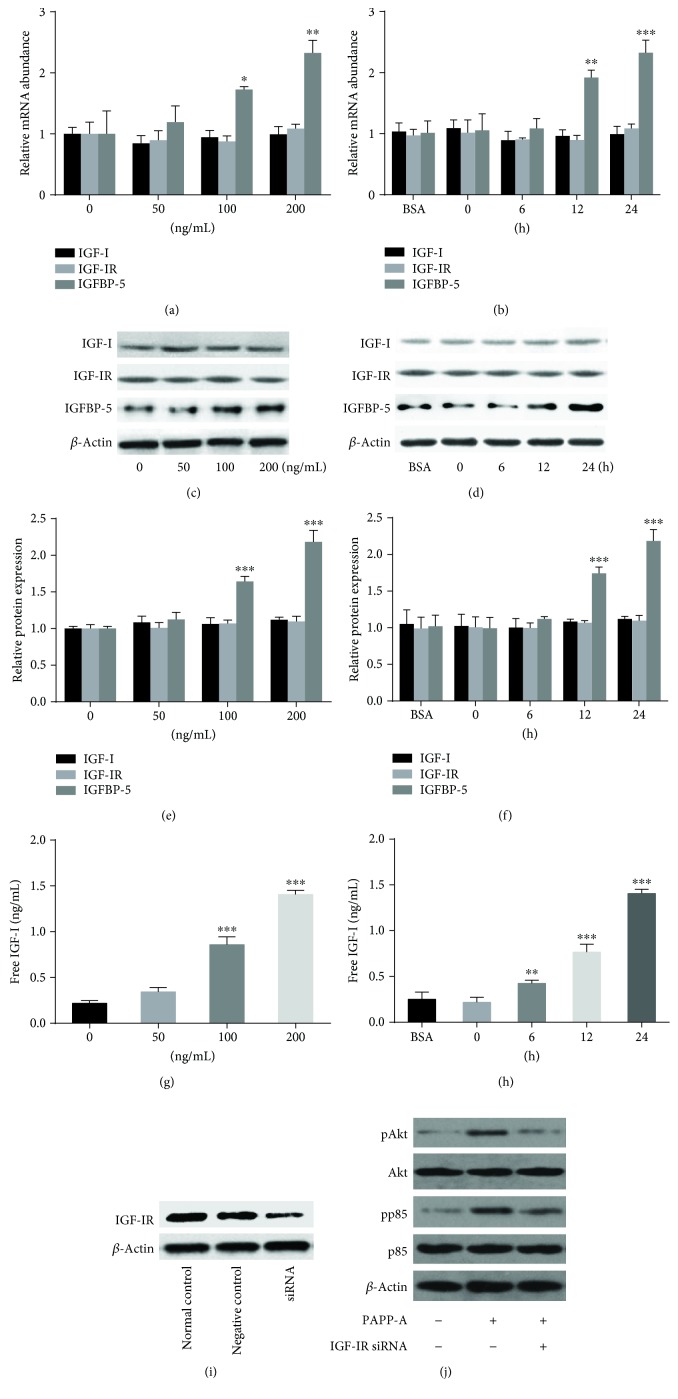
PAPP-A induces the IGF-I/PI3-K/Akt pathway in macrophages. (a, c, e) RAW264.7 macrophages were treated with various concentrations of PAPP-A for 24 h, respectively. (b, d, f) Cells were treated with 5 mg/mL BSA for 24 h or with 200 ng/mL PAPP-A for different durations, respectively. (a, b) IGF-I, IGF-IR, and IGFBP-5 mRNA expressions were measured by RT-qPCR. (c, d, e, f) IGF-I, IGF-IR, and IGFBP-5 protein expressions were measured by Western immunoblotting assays. (g, h) Free IGF-I concentrations in the culture medium was estimated with ELISA. (i, j) RAW264.7 macrophages were transfected with control or IGF-IR siRNA and then incubated with PAPP-A (200 ng/mL) for 24 h. (i) Protein samples were immunoblotted with anti-IGF-IR or anti-*β*-actin antibodies. (j) Western analysis for phosphorylated Akt and PI3-K p85 subunit. Similar results were obtained in three independent experiments. Data are the mean + SD. ^∗^*P* < 0.05 vs. baseline, ^∗∗^*P* < 0.01 vs. baseline, and ^∗∗^*P* < 0.001 vs. baseline.

As shown in Figure 3, RT-qPCR analysis revealed that PAPP-A did not affect the level of IGF-I and IGF-IR mRNA. However, PAPP-A increased IGFBP-5 mRNA and protein levels in a dose-dependent and time-dependent manner. Furthermore, to exclude the possibility that an increase in the free IGF-I level is due to increased IGF-I production in response to PAPP-A treatment [21], we measured the free IGF-I in the culture supernatant. The free IGF-I level was very low under BSA or basal condition. PAPP-A significantly increased the free IGF-I levels and showed half maximal effectiveness at approximately 100 ng/mL. In addition, free IGF-I concentrations increased 6 hours after stimulation with PAPP-A (200 ng/mL) and continued to elevate by approximately 7-fold at 24 h (Figures 3(g) and 3(h)). These results indicate that PAPP-A may increase free IGF-I production and IGF-I bioavailability. On the other hand, we observed that PAPP-A increased the phosphorylation of Akt and of the p85 regulatory subunit of PI3K in RAW264.7 macrophages (Figure 3(j)). After 48 hours of the transfection, in comparison to the control siRNA, the siRNA of IGF-IR suppressed the expression of IGF-IR proteins by 80% according to Western blot analysis (Figure 3(i)), suggesting the effectiveness of our IGF-IR siRNA. The PAPP-A-mediated activation of the PI3K and Akt was significantly blocked by IGF-IR siRNA (Figure 3(j)). All of our results suggest that PAPP-A can activate the IGF-I/PI3K/Akt pathway in RAW264.7 macrophages.

### 3.4. IGF-I/PI3K/Akt Signaling Pathway Was Related to PAPP-A-Mediated Upregulation of MCP-1, TNF-*α*, and IL-6

**Figure 4:**
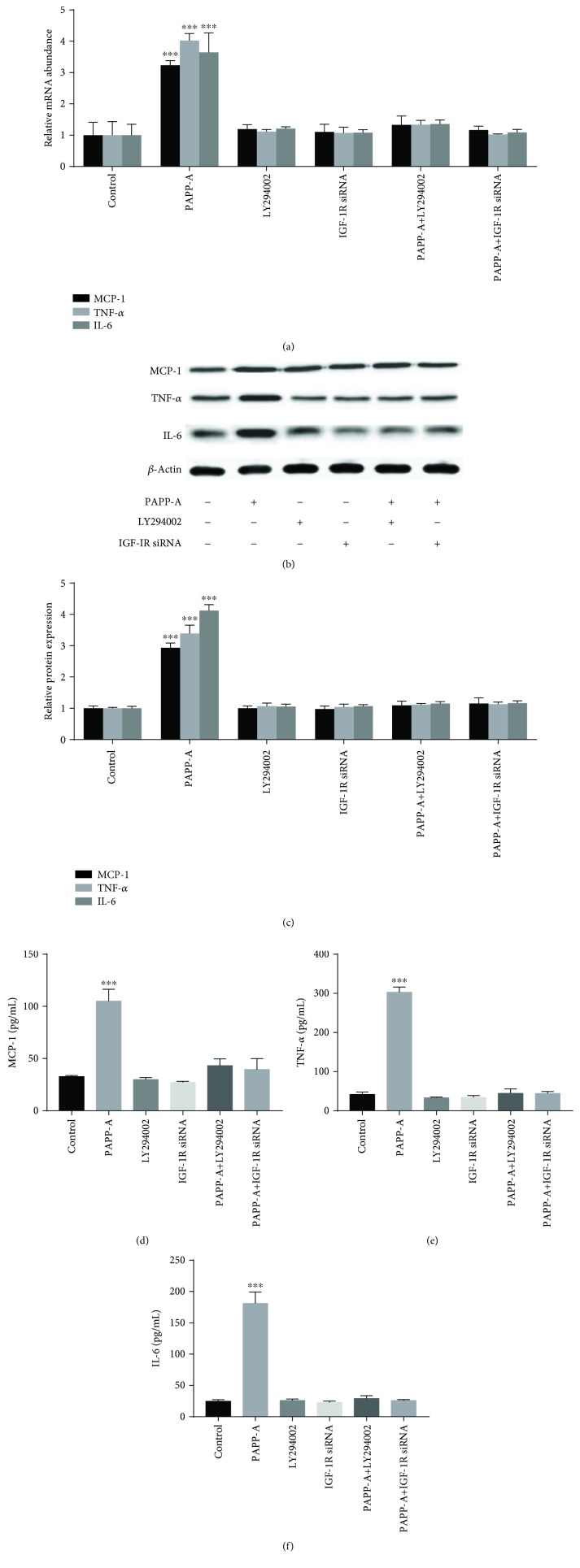
Upregulation of MCP-1, TNF-*α*, and IL-6 expressions and productions by PAPP-A is dependent on the IGF-I/PI3-K/Akt signaling pathway in RAW264.7 macrophages. Cells were incubated for 24 h in the presence (+) or absence (−) of PAPP-A (200 ng/mL) with (+) and without (−) LY294002 (10 *μ*M). In addition, RAW264.7 macrophages were transfected with control or IGF-IR siRNA and then incubated with PAPP-A (200 ng/mL) for 24 h. Total RNA was extracted, and RT-qPCR was performed to determine the expression of MCP-1, TNF-*α*, and IL-6 mRNA (A), and Western immunoblotting assays were conducted for protein expression of MCP-1, TNF-*α*, IL-6, and *β*-actin (b, c), and then ELISA were used to estimate MCP-1, TNF-*α*, and IL-6 concentrations in the culture medium (d, e, f). Similar results were obtained in three independent experiments. Data are the mean + SD. ^∗∗∗^*P* < 0.001 vs. baseline.

Previous research revealed that IGF-I can induce the PI3K/Akt signaling pathway in macrophages. To determine if the PAPP-A-mediated increases in the expression and production of MCP-1, TNF-*α*, and IL-6 were associated with PI3K/Akt pathways, we performed RT-qPCR, Western blot, and ELISA in the absence or presence of LY294002 (10 *μ*M), a selective inhibitor of the PI3K/Akt signaling pathway, in RAW264.7 macrophages. As shown in Figure 4, LY294002 pretreatment blocked the PAPP-A-mediated upregulations of MCP-1, TNF-*α*, and IL-6 mRNAs (Figure 4(a)), proteins (Figures 4(b) and 4(c)), and secretion (Figures 4(d)–4(f)). On the other hand, the same effects were also observed by using IGF-IR siRNA. Treatment with IGF-IR siRNA reversed the upregulation of PAPP-A on MCP-1, TNF-*α*, and IL-6 expression and secretion (Figure 4). These results indicate that PAPP-A may upregulate MCP-1, TNF-*α*, and IL-6 expressions by activating the IGF-I/PI3K/Akt signaling pathway.

## 4. Discussion

Our study showed that PAPP-A increased the expression and production of MCP-1, TNF-*α*, and IL-6 by stimulating the IGF-I/PI3K/Akt signaling pathway in RAW264.7 macrophages. Our findings support the notion that PAPP-A may contribute to plaque formation and rupture in atherosclerosis by promoting inflammation.

Previous studies from our group and other investigators have indicated that PAPP-A might be a marker for ACS and could predict clinical outcomes [3, 8, 11, 22, 23]. A meta-analysis has also demonstrated that an elevated PAPP-A level was positively associated with cardiovascular events, and this positive association was not affected by follow-up duration, coronary artery disease type, and different measurements of PAPP-A [24]. Several animal model studies have indicated that PAPP-A might be involved in atherosclerotic lesion development. PAPP-A KO mice showed little or no atherosclerotic lesion development. PAPP-A KO/ApoE KO fed a high-fat diet had the same number of lesions compared with ApoE KO mice but had 60-80% reduction in an aortic lesion area [11]. On the other hand, transgenic overexpression of PAPPA expression accelerated atherosclerotic plaque development and complexity in ApoE KO mice [15, 25]. In an ApoE-null mouse model, an inducible decrease in PAPP-A gene expression significantly inhibited atherosclerotic plaque progression as assessed by a 70% reduction in plaque burden in the aorta and prevented the development of advanced plaque with necrotic cores and buried fibrous caps in the brachiocephalic artery [14]. Recently, Conover et al. reported that an inhibitory monoclonal antibody (mAb-PA) reduced aortic plaque burden and plaque complexity in ApoE KO mice on a high-fat diet; i.e., plaques appeared as early-stage fatty streaks whereas the control plaques showed multiple acellular necrotic cores. This is the first study to demonstrate the proof of principle and provide feasibility for a novel therapeutic strategy to inhibit atherosclerotic plaque burden by selectively targeting PAPP-A [13]. These findings suggest PAPP-A not only is a serum biomarker of ACS but also is involved in the pathophysiology of ACS.

Bayes-Genis et al. first found PAPP-A in the inflammatory shoulder region, the areas surrounding the lipid core, smooth muscle cells, and the extracellular matrix [3]. A study has found that a higher PAPP-A level was associated with higher 3-vessel thin-cap fibroatheroma burden in patients with coronary artery disease [26]. In addition, PAPP-A has been detected mainly in inflammatory cells, such as monocytes and macrophages, when examining carotid plaques [5]. Increased PAPP-A activity can increase lipid accumulation and impair cholesterol efflux from macrophages via negative regulation of ABCA-1, ABCG-1, and SR-B1 associated with activated macrophages and smooth muscle cells [27, 28]. In our previous study, we found that PAPP-A was a stronger predictor of the increased risk for future cardiovascular events in patients with ACS [8]. Additionally, PAPP-A expression and production can be induced by CRP through the NF-*κ*B-dependent pathway in human peripheral blood mononuclear cells, and this mechanism may play a critical role in increasing serum PAPPA levels during ACS [29].

Inflammation and immune activation are involved in ACS pathogenesis. Macrophages play a critical role in the initiation and maintenance of inflammatory response. They can be activated by numerous soluble stimuli, including lipopolysaccharide (LPS), interferon-*γ* (IFN-*γ*), granulocyte colony-stimulating factor (GCSF), and extracellular matrix protein [30]. In inflammation response, activated macrophages will release a large number of chemokines and proinflammatory cytokines, which will further exaggerate the inflammation response, ultimately leading to a “cascade” type of inflammation responses [31, 32]. MCP-1 is the prototype of the C-C chemokine *β* subfamily and exhibits its most potent activity on monocytes. It rapidly causes rolling monocytes to adhere firmly onto endothelial cells expressing E-selectin and monocyte infiltration into the subendothelial space. Besides monocyte recruitment, MCP-1 activates monocytes and induces the expression of tissue factor (TF), superoxide anions, and proinflammatory genes [33, 34]. TNF-*α*, a potent pleiotropic cytokine, is mainly produced by the activated macrophages in the atheromatous plaque. It exerts proinflammatory effects and promotes the uptake of oxidized LDL by macrophages, foam cell formation, leukocyte recruitment, and endothelial dysfunction. TNF-*α* may also contribute to plaque destabilization and acute thrombosis [35]. In vitro studies on human macrophages have shown that TNF-*α* increased the production of matrix-degrading metalloproteinases [36]. IL-6, similar to TNF-*α*, is also an important proinflammatory cytokine and secreted by many cell types, including monocytes and macrophages. It induces atherosclerosis locally by inducing inflammation at a plaque site and systemically via the hepatic acute-phase reaction, which involves CRP, angiotensinogen, fibrinogen, and complement components [34, 37]. In addition, via activation of the macrophages, IL-6 increases matrix metalloproteinase expression, hence accelerating degradation of the extracellular matrix [38]. Up to now, it has not been reported whether PAPP-A can directly induce inflammatory cytokines and chemokines. In the current study, PAPP-A stimulated RAW264.7 cells to increase MCP-1, TNF-*α*, and IL-6 expressions and productions, indicating that macrophages may be activated by PAPP-A. These results indicate that PAPP-A may cause or enhance inflammation by activating macrophages, which consequently may increase plaque vulnerability.

**Figure 5:**
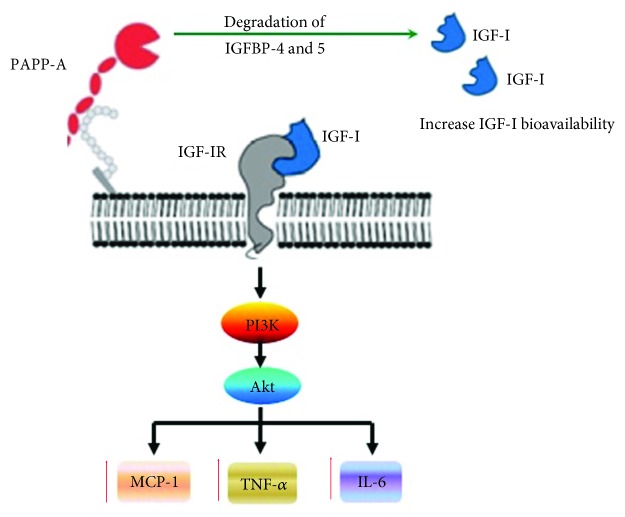
Schematic representation of the induction of MCP-1, TNF-*α*, and IL-6 expressions by PAPP-A through the IGF-I/PI3K/Akt pathways in RAW264.7 macrophages.

In the present study, we observed that PAPP-A increased IGFBP-5 expression levels in a dose-dependent and time-dependent manner in RAW264.7 macrophages and significantly increased free IGF-I levels. We also found that PAPP-A increased the phosphorylation of Akt and of p85 regulatory subunit of PI3K. Our results showed that IGF-IR siRNA blocked PAPP-A-mediated upregulation of Akt and PI3K phosphorylation in RAW264.7 macrophages. Therefore, our study supports that PAPP-A can upregulate and activate the IGF-I/PI3K/Akt pathway in RAW264.7 macrophages. Moreover, we tested the effects of the PI3K inhibitor LY294002 on the expression profile of inflammatory cytokine in RAW264.7 macrophages. Our results indicate that the increased levels observed in MCP-1, TNF-*α*, and IL-6 mRNA and protein levels upon treating cells with PAPP-A were strongly inhibited with LY294002. Furthermore, the treatment with siRNA for IGF-IR downregulated IGF-IR protein expression by 80% and completely reversed the effects of PAPP-A. Hence, we concluded that the upregulation of these inflammatory cytokines induced by PAPP-A was mediated by the IGF-I/PI3K/Akt signaling pathway (Figure 5).

In conclusion, we present in vitro evidence that PAPP-A induced MCP-1, TNF-*α*, and IL-6 expressions and productions in RAW264.7 macrophages by activating the IGF-I/PI3K/Akt pathway. This proinflammatory mechanism may contribute to plaque rupture and the consequent ACS. Our findings indicate that PAPP-A could be a novel therapeutic target to inhibit atherosclerotic plaque progression and prevent the occurrence of ACS.

## Figures and Tables

**Table 1:** Primers used for the quantitative real-time PCR.

Primers	GenBank number	Sequence (5′-3′)	Size (bp)
*β*-Actin	NM_007393.5	F: CGTTGACATCCGTAAAGACC	111
		R: CTAGGAGCCAGAGCAGTAATC	
MCP-1	NM_011333.3	F: CCCTAAGGTCTTCAGCACCT	118
		R: ACTGTCACATGGTCACTCC	
TNF-*α*	NM_013693.3	F: GTGCCTATGTCTCAGCCTCT	129
		R: CTGATGAGAGGGAGGCCATT	
IL-6	NM_031168	F: AGACTTCCATCCAGTTGCCT	113
		R: CAGGTCTGTTGGGAGTGGTA	
IGF-I	NM_010512.5	F: ACTGGAGATGTACTGTGCCC	105
		R: GATAGGGACGGGGACTTCTG	
IGF-IR	NM_010513	F: TCCCCAACCTCACAGTCATC	146
		R: GTCGGCGTTCTTCTCAATCC	
IGFBP-5	NM_010518	F: CGAGATGGCTGAAGAGACCT	112
		R: CTGGGTCAGCTTCTTTCTGC	

MCP-1: monocyte chemoattractant protein-1; TNF-*α*: tumor necrosis factor-*α*; IL-6: interleukin-6; IGF-I: insulin-like growth factor-I; IGF-IR: insulin-like growth factor-I receptor; IGFBP-5: insulin-like growth factor binding protein-5.

## Data Availability

The data used to support the findings of this study are included within the article.
